# Marine spatial planning: Coordinating divergent marine interests

**DOI:** 10.1007/s13280-020-01471-0

**Published:** 2021-02-07

**Authors:** Kjell Grip, Sven Blomqvist

**Affiliations:** 1 Department of Ecology, Environment and Plant Sciences, Stockholm University, 106 91, Stockholm, Sweden; 2 Present Address: Mandelblomsgatan 11, 745 36 Enköping, Sweden

**Keywords:** Ecological approach, Ecosystem-based, Marine management, Multi-sectoral and adaptive, Sectoral coordination, Spatial planning

## Abstract

Globally, ecosystem-based marine spatial planning has become a useful instrument to coordinate the planning of different authorities. This, for balancing different requirements when managing marine areas and space. In the planning process, ecology is setting limits to which human activities are acceptable to the society. The use of the marine environment can be planned similarly as the land environment. We argue that there are several aspects which must be taken into consideration. Marine activities have traditionally been planned and managed in a sectoral way. Today, it has become obvious that a more holistic, multi-sectoral and coordinated approach is needed in future successful marine planning and management. The increased awareness of the importance of the oceans and seas challenges the traditional sector division and geographical limits in marine policy and calls for better coordinated and coherent marine policies.

## Introduction

Already in the 1970s, the use of Marine Spatial Planning (MSP) was discussed in the marine administration of several European countries. In 1985, the Council of Europe held a seminar on *The Development and Planning of Coastal Regions* (CDAT 1985). The ecological aspects of marine spatial development were a theme in the seminar. However, it was not until the early 2000s, and later, that this form of planning really began to take off, likely as a result of the first international workshop on MSP organized by UNESCO/IOC in 2006.

Worldwide, in many different countries, the evolution of MSP, involving ocean zoning (Agardy 2015; Ehler 2017; Santos et al. 2019), has become a crucial step in making a multi-sectoral and ecosystem-based sea use planning and management a reality (Douvere 2008; Douvere and Ehler 2009). The idea was initially stimulated by international and national interests for improving multiple use management of Marine Protected Areas, for example, in the Great Barrier Reef Park in Australia (Day 2002). Also, the role of society during the planning phase includes permit applications in accordance with a wide range of laws relating to changes and activities, with effect on land and water areas. In that respect, MSP is a form of policy-making.

Maritime^1^ or Marine Spatial Planning is commonly defined as “a process of public authorities of analyzing and allocating the spatial and temporal distribution of human activities in marine areas and space to achieve ecological, economic and social objectives. It is an instrument for reducing conflicts and strengthen the coordination between sectors and protect the environment through assessing the environmental effects of concerned activities” (EU 2011).

Urban expansion and coastal developments, pollution from industries and rivers, fishery, aquaculture, renewable energy, mineral extraction, military defense, recreational boating, outdoor recreation and nature conservancy are examples of interests that are related in one way or another to the utilization and protection of coastal and marine resources. All of them affect coastal waters and marine environments in some way.

Conflicts between the various interests and activities in and above a water body are common. With increased use of marine resources this has resulted in demands for a better planned use and protection of the marine environment and its resources (DSH 1989; Ackefors and Grip 1995; Douvere and Ehler 2009; UN Environment 2018). However, methods for addressing various aspects of physical and social planning in our coastal and marine areas have been, and are still partially undeveloped or new legal practices have not yet been implemented or enforced (OECD 2017; Ehler et al. 2019). The use of MSP is more prominent in developed than developing countries (Pomeroy et al. 2014).

There are a number of different forms of planning related to MSP, and practice varies from country to country, due to different geographical conditions, marine pressures, economic disparities, legal requirements and planning cultures (EUCC 2002; UN Environment 2018). Few countries have specific legislation for marine spatial planning. Existing legal instruments are used to regulate and implement different marine policies through MSP.

In Germany, for example, the competence for MSP inside the territorial boundary is linked to land planning and rests by the coastal states. In the Exclusive Economic Zone, the competence is federal and there are spatial plans with federal legal ordinances. The Netherlands has a National Water Plan, coordinated by an interdepartmental committee. Outside 1 km from the coastline, the central government is the only competent authority and responsible for the North Sea policy. The European MSP Directive (Directive 2014/89/EU) requires all coastal Member States to prepare cross-sectoral maritime spatial plans by 2021 with societally agreed goals, values and targets. These plans can include permits and other administrative decisions, but also different non-binding visions, strategies and governance principles related to the usage of the sea (OECD 2017).

The marine environment can be planned in the same way as the land environment (DSH 1989; Grip 1992; Kidd and Ellis 2012). Already in the 1980s, Swedish coastal and marine management studies demonstrated that there was no need for a special type of water use planning. However, there are several additional aspects which must be taken into consideration in the comprehensive physical planning of water resources, not least in relation to multinational jurisdiction and cooperation through global and regional conventions and agreements. Also, consideration of ecological, social and economic concerns in connection with the use, protection and management of surface water areas, water volumes, the seabed and the air space above the surface are fairly recent. However, the MSP and decision-making process for managing these areas follow the same planning steps (Santos et al. 2019). Also, the generality of planning gives it a wide applicability (Faludi 1973).

Another important aspect is the relationship between measures enacted on land and in coastal and marine waters. Many environmental problems in such waters have their origin in land activities. For this reason, the MSP process needs to be adapted to include the use and protection of both land and water resources (Grip 1992; Scholten et al. 2019).

The increased utilization of coastal and marine resources has strengthened the need for an environmentally, technologically and socio-economically sound management of coastal and marine areas (UNEP 1996; Grip 2017; Santos et al. 2019). For this to function, the MSP administration needs to be linked to both research and an environmental monitoring, designed as a central support for follow-up and evaluation of the measures taken.

The overall aim of the present paper is to review the development and practical use of MSP as a tool for coordinating different marine activities. This, to balance the use and protection of marine areas with their resources, biodiversity and ecosystem services.

Among the world’s coastal countries there are several different approaches to MSP (Santos et al. 2019). In our study, older and newer literature related to spatial planning worldwide have been reviewed. The main sources are scientific journals and public planning publications in Europe and elsewhere. Various databases accessible through the Internet and at university libraries have been used. Also, personal experiences of marine spatial planning in practice, of one of us (KG), at the Swedish Environmental Protection Agency and in several international environmental organizations underlie the article.

## Physical planning: A supporting tool in coastal and marine management

Coastal and marine management is a collective term for the administrative procedures for MSP employed by central, regional or local governments in most countries. It is an interdisciplinary field that requires an understanding of the marine environment and its resources, but also of economic, social, technical, legal and administrative conditions related to the sea (Douvere 2008; Ehler and Douvere 2009). The management is governed by the marine policy of a country, which is usually delegated to one or several sectoral ministries of Government.

Recently, spatial planning for the coordination of more offshore marine activities has become common. However, the utilization of natural resources in coastal and marine areas is still mainly dealt with on a sectoral basis, and is usually driven by commercial interests (Johannesen and Lassen 2014) (Box 1). Examples of such utilization are allocation of fishing quotas, extraction of sand and gravel, and planning of shipping lanes and wind parks.

The need for national governance of principles and overarching issues has been pointed out by various marine actors. This is especially true for problems in coastal zones that are linked to safety at sea, protection of the environment and regional development. In such cases, a multi-sectoral MSP process, involving the coordination of all concerned interests, has become an important tool in coastal and marine management (White et al. 2012; Smythe 2017).

**Box 1:** Sectoral versus coordinated or integrated planning and management

Marine activities have traditionally been planned and managed in a sectoral way, backed up by sectoral ministries, agencies, non-governmental organizations and sectoral legislation. Conflicts between interests are common and often rooted in disagreements between the responsible ministries and the contention surrounding political determination. The MSP process is a multi-sectoral process, and based on a coordination or, if possible, integration of all concerned activities and interests
Integrated management of different activities implies a departure from sectoral planning towards an inter-sectoral approach (Stead and Meijers 2009). Also, integration imply detailed engagement with social and political processes to bring greater coherence in decision-making. However, integration is difficult to achieve, since each agency usually finds it easier to work on a sectoral or separate basis, as you need to consider legal aspects other than those you represent (Dodds et al. 2012). It is easier to coordinate work between sectors. Integration requires changes in the organizational structure, while coordination does not change the internal structure of an organization

### Internationally Marine Spatial Planning practices are different

The use and protection of coastal and marine areas also involve multinational jurisdiction and raise the issue of international cooperation by both governmental and non-governmental organizations. The international exchange of information is of great significance to coastal and marine management, particularly in the environmental field. International cooperation is established through several global and regional conventions, agreements and programmes, for example, the United Nations Convention on Law of the Sea (UNCLOS), MARPOL 73/78, the UN Convention on Biological Diversity, the FAO Regional Fishery Commissions, the UNEP Regional Seas Programme, the Helsinki and OSPAR Conventions, and others.

As mentioned, planning and management practices differ among countries (EUCC 2002). This influences the implementation and enforcement of recommended or decided agreements. To be efficient the MSP process must be enshrined in national legislation. In the MSP process, states can propose restrictions on uses of waters under their jurisdiction and sovereignty, to protect sensitive ecosystems, including restrictions on certain types of uses in designated areas (EU 2011; UN Environment 2018). This process requires participation of all concerned stakeholder groups. Also, the public sector increasingly relies on the MSP process, not least in planning of human settlements and natural resources management. In the MSP process, stakeholders and non-governmental organizations should have the right to comment on how the authorities are performing their obligations. Involving stakeholder knowledge, views and needs in the MSP processes is important from a governance and social sustainability perspective.

In 2015, the *2030 Agenda* with its Sustainable Development Goals was adopted, aiming at a better and more sustainable future for all. Goal 14: *Conserve and sustainably use the oceans*, *seas and marine resources* stresses the importance of healthy oceans for sustainable development and demands careful planning and management of the oceans and seas. To strengthen the work on the development goal, the United Nations (UNESCO/IOC) has announced a *Decade of Ocean Science for Sustainable Development* (*2021–2030*) (IOC/BRO/2018/2).

A well-functioning MSP process is needed to implement the EU’s *Integrated Maritime Policy* (The European Commission, COM/2008/0395 final) and to stimulate so called “blue growth”. The main coordination instrument for this policy is the *Framework Directive for Maritime Spatial Planning* (Directive 2014/89/EU). It should be noted that there is a potential conflict between the EU’s maritime policy on blue economic growth and the objectives of the *Marine Directive* (Directive 754 2008/56/EC) regarding the concept of Good Environmental Status. The EU Maritime Policy promotes the economic use of the sea, which may lead to conflicts with the aim of preserving a Good Environmental Status according to the Marine Directive.

A corresponding policy is found in the *UN Blue Economy Concept* (UNEP 2012; Ehler 2017). Recently, UNESCO’s Intergovernmental Oceanographic Commission (IOC-UNESCO) and the European Commission (Directorate General for Maritime Affairs and Fisheries of the European Commission) have launched *MSPglobal*, a new joint initiative to promote cross-border maritime spatial planning in support of Goal 14 of the Agenda 2030 on Sustainable Development. This initiative was a result of the second international workshop on MSP organized by IOC/UNESCO in 2017 (Ehler 2017).

The Agenda 2030 target for Marine Spatial Planning is that ≥ 33% of the surface area of world’s exclusive economic zones under national jurisdiction should have approved plans by 2030 (Ehler 2017; MSPglobal 2017).

Furthermore, a multi-sectoral and ecosystem-based MSP process should, with some complements, be applicable also in the High Seas, including the seabed and the air space above the surface, provided that the lack of regulation under the UNCLOS is addressed^2^ (Ardron et al. 2008; Houghton 2014). This regulation should include an appointment of a responsible UN programme.^3^ It should be noted that five Regional Seas Conventions, within and outside the UN, already include Areas Beyond National Jurisdiction within their geographical coverages, for example, the OSPAR and Barcelona Conventions for the North-east Atlantic and the Mediterranean Sea, respectively (UN 2017). However, this jurisdiction only applies to their contracting parties.

### What is Marine Spatial Planning?

Sector-based planning according to sectoral laws has the sectoral interest in focus. Multi-sectoral spatial planning according to spatial planning law is an activity aimed at determining how land and water areas and space are to be utilized for different purposes in time as well as in space. It involves trying to see things wholly and setting goals, and trying to figure out the best way of achieving them (Perloff 1957). In that respect, MSP involves coordination with other planning frameworks, related to legal, socio-economic and environmental interests (OECD 2017). Usually, the result of the multi-sectoral planning process is an overall plan with regulation of measures and management guidelines in order to ensure that vital societal interests are taken into consideration (Ehler 2017; Santos et al. 2019).

Reliable comprehensive spatial planning is a fundamental requirement in current marine management, in addition to sectoral planning and management. Critical, scientific studies are crucial as support for responsible spatial planning, and to balance the claims of different interests. This has long been recognized (Perloff 1957; Beer 1966; Quade 1968; Faludi 1973), and has recently been reiterated (Guerry et al. 2012).

To get an overview of MSP is difficult, because the planning is mainly operated as sectoral planning by sector-bound expertise. The comprehensive spatial planning process, according to an MSP law, involves the coordination of different sectoral interests (Box 2). However, sectoral laws and overlapping national and international legislation affect and complicate the legal review of various activities in the planning process. Planning is and should be “a general approach to decision-making and is not tied to the activities of any profession or department of government” (Faludi 1973).

**Box 2:** Outline of an MSP process

The MSP process for a certain area involves all concerned interests and their stakeholders (Ehler and Douvere 2009). Such interests are subject to the planning, granting of permits and other controls exercised by government authorities. Each sector provides information about its activities related to practical, technical, socio-economic, environmental and legal issues. Scientific analysis will make the conflicts clear and may help the involved parties in understanding where the objective conflicts are and how the technical measures that are proposed together with compromise proposals will influence their position. Also, enlarging the scope of the underlying analysis helps to strengthen the arguments brought to the negotiating table
Among the interests involved in the process some may be collaborative others are in conflict. The former is seldom a problem in the planning and decision-making process. It is the conflicting interests that are the focus of the process—how these interests can be coordinated or integrated. Sometimes consensus can be reached but when that is not the case, there need to be one agency with the overall mandate and responsibility for the sectoral coordination and to take the final decision, or transfer the case for decision to a higher level. In most cases such a decision, in the form of a plan with guidelines and recommendations, is accepted but it does not mean that it is well received by all stakeholders. If an MSP process is not in place, sector-based management applies. It means that it is the sector with the strongest legislation that decide, and sector-based decisions often contradict the required decisions in other sectors

### Deficiencies in marine spatial planning

MSP has become a rather common decision support instrument, but has deficiencies and potential negative effects of its use, for example, related to negotiations on the use of fish and ecosystem issues—who is a legitimate stakeholder and which criteria for good governance shall be met? This has been addressed in some recent contributions (Johannesen and Lassen 2014; Flannery and Ellis 2016; Janßen et al. 2018). Flannery and Ellis (2016) call for an MSP process with “a more equity-based democratic decision-making, and a fairer distribution of marine resources”. Other aspects such as consideration to ecosystem services have been proposed to facilitate the dialogue and to inform the decision-making process (Guerry et al. 2012). The planning itself ought to be understood as a rational problem-solving strategy employed in a particular social and institutional context (Faludi 1973). However, there are various beliefs about planning, about opportunities to plan and about democracy in the planning process (Ehler et al. 2019).

Physical planning of marine areas is not different from planning of land areas. The MSP field is dominated by natural scientists, and usually their expertise is not associated with planning. However, MSP as an interdisciplinary field would benefit from more educated planners involved in the planning process. Professional planners could contribute to and improve the MSP process, for example, related to involvement of stakeholders, planning methods, transboundary planning, goal-setting, coordinating different perspectives into goals, and integrating land-use and marine planning objectives with terrestrial planning (Kidd and Shaw 2014; Retzlaff and LeBleu 2018).

Despite plenty of research related to MSP (Douvere and Ehler 2009), there are still major shortcomings in practical marine environmental governance and management. For example, in the coordination of natural and social scientific perspectives in the overall MSP process, you need to consider the effects of human usage on key ecosystem processes, such as population connectivity, interaction webs and biogeochemistry (Crowder and Norse 2008). Also, social, cultural, economic, institutional, legal and political aspects overlay the biophysiological aspects. This is shown, for example, when stakeholders participate in an MSP process about the establishment of a marine protected area or a wind park (Reed 2008; Gall and Rodwell 2016; Hassler et al. 2018).

According to UNCLOS, MSP is legally a national issue, where the governance and management of sea activities within national jurisdiction rests at the government or an agency mandated by the government. However, several issues are cross-boundary. Usually, countries do cooperate on cross-border issues through international organizations such as HELCOM, OSPAR and UNEP Regional Seas. Still, the responsibility rests by the government. This may complicate and delay the planning and decision-making process in cross-boundary issues. An example is the complex cross-boundary conflict, between fishery and the EU`s protected area Natura 2000, at Dogger Bank in the North Sea (Johannesen and Lassen 2014).

Currently it is argued that cross-boundary issues cannot be addressed properly on the basis of national concerns alone. As Johannesen and Lassen (2014) points out, governments are not inclined to abdicate responsibility for cross-border issues. Some argue that the regional commissions such as HELCOM and OSPAR should be given a stronger role in the MSP process. However, the responsibility rests by the government and governments are not yet willing to hand over their responsibilities to an international body. The EU’s MSP Directive stipulates that Member States should ensure transboundary cooperation between Member States, as well as to promote cooperation with third countries.

### An efficient marine spatial planning process

Previous investigation of marine spatial planning and decision-making has been limited. Examination of prior efforts shows strict respect for the limiting boundaries of disciplinary traditions (e.g. fishery) and lack of international perspective. Little attention has been given to exploring the potential contribution from relevant social and economic sciences, and the long-term effects of the used planning process for the decision-making. To be efficient the future planning practices related to different marine users and activities need to be more adapted to the socio-economic and political culture in which it operates, while taking into account the spatial and temporal diversity of the marine environment (Douvere 2010).

Such a well-established integrated planning and decision-making process based on natural and socio-economic analyses will have long-term effects on economic as well as ecological and socio-cultural values in the sea. It should be noted that the implementation of all plan-based approaches requires progress reviews and needs to be revised at certain intervals depending on the societal changes that are taking place.

### What is planned?

It is important to be aware of what kind of planning is conducted. “Real” planning requires that the planner also controls the resources to be planned (Björkman et al. 1976). In Sweden and other market economy countries, the municipalities and the state control only a very small part of the real physical and financial resources. In addition, international regulations need to be considered. Neither the municipalities nor the state can plan the use of land and water resources as they want to. In marine spatial planning, it is the state that has the priority of interpretation and state planning is related to policy and mostly defensive. The actual planning that is really produced is a kind of “adaptive” planning that needs to consider, or have an adaptive approach to other societal interests and relevant regulations (Douvere and Ehler 2010).

The United Nations Convention on Law of the Sea, called *The Constitution of the Sea*, entered into force in 1994. According to UNCLOS, the state usually controls the use of marine resources under its sovereignty and responsibility, unlike the situation on land. Provided international conventions and agreements are respected, and that there is a political will, marine spatial planning should be a “real” planning, where the “property owner”, the state, decides how the marine resources should be used (Grip and Blomqvist 2020).

### The ecological approach and forcing principles in marine spatial planning

**Fig. 1:**
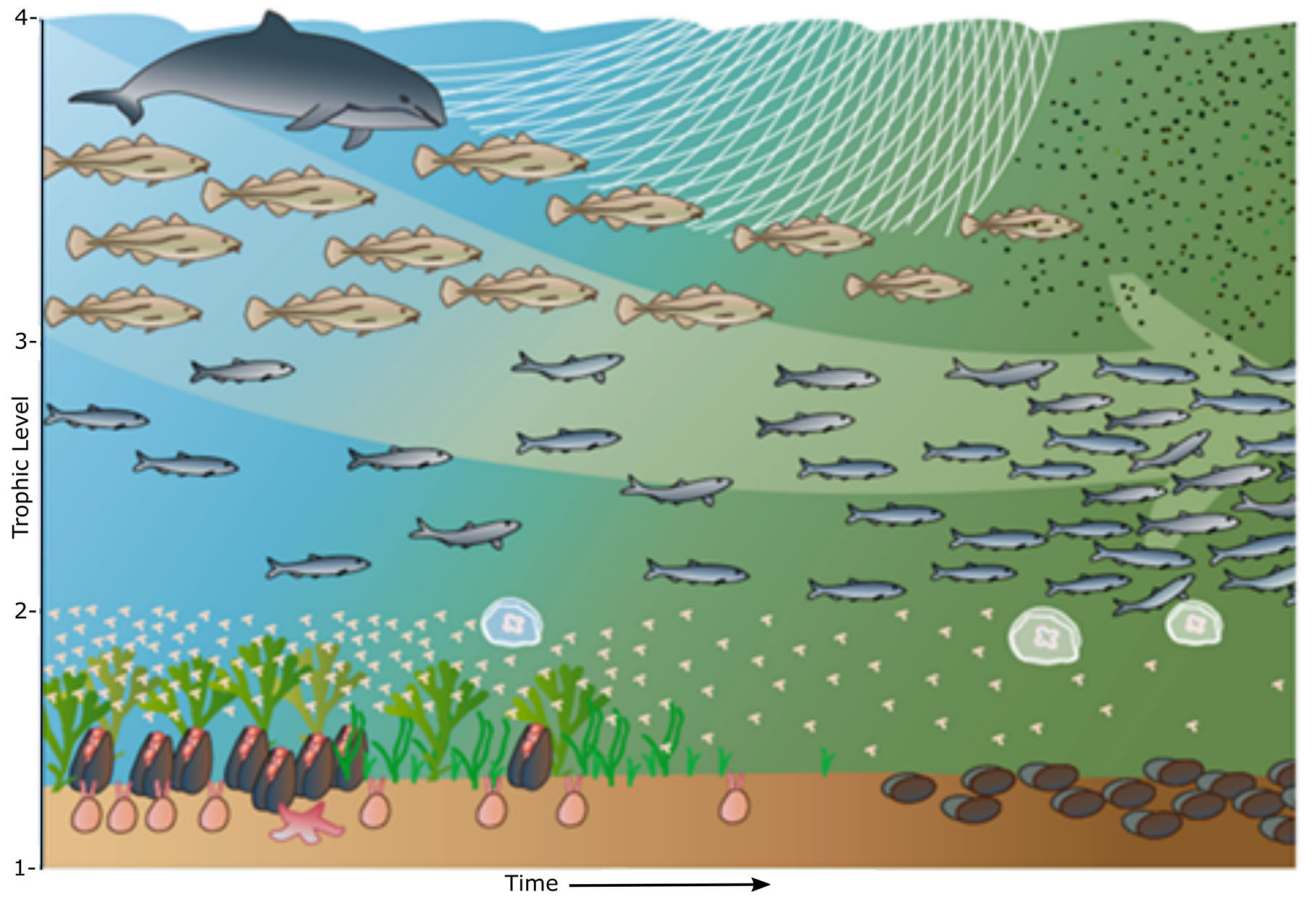
Ecological effects of eutrophication and over-fishing in the Baltic Sea, illustrated as changes in the food web structure. The figure shows changes in trophic levels over time, from complex food webs to food webs with low biodiversity and simple functionality. This as a consequence of human activities. The ecosystem effects of an activity need to be considered as a basis for the MSP process. *Source* Nordic Council of Ministers, Copenhagen, Denmark (Belgrano 2018)

Ecology has long been able to contribute to a variety of important social issues, such as: How does the marine environment with its species and habitats function? How should we behave to get the human relationship to the natural environment and ecosystem services to work? (Flannery and Ellis 2016). An ecological approach reflects the need to understand the very different environmental consequences of human activities (Fig. 1). That ecology is setting limits to which human activities are acceptable to the society.

In 1972, the concept of an *ecological approach* was introduced as the basis for the Swedish National Physical Planning system by the Swedish Parliament (Carlberg and Grip 1982; Grip 1992). It was based on the principle “that prior to decisions that cause an intrusion on the environment, the environmental effects of the decision, as well as the current environmental status of the affected land or water area should be known”.

Today, the ecological approach has been subsumed under instruments such as the ecosystem approach, ecosystem-based management (Browman et al. 2004; Crowder and Norse 2008; Ehler and Douvere 2009) and other instruments related to MSP (Cicin-Sain and Belfiore 2005; Guerry et al. 2012). Most of these instruments focus on the environmental aspect of the physical planning process and they should be seen as instruments supporting the overall MSP process (Box 3). It is the multi-sectoral MSP process that should be ecosystem-based in support of the decision-making in the marine administration. In a well-implemented MSP process, consideration is given to how human activities affect the ecosystems, while also taking socio-economic conditions into account (Domínguez-Tejo et al. 2016).

**Box 3:** Different approaches to ecosystem-based planning and management

The ecosystem approach is often used synonymously with ecosystem-based management and other similar concepts. There are many ways in which these concepts can be used. According to Murawski (2007) “The ecosystem approach is generally defined as extending existing management foci (e.g., fisheries) to include additional considerations consistent with ecosystem management characteristics, while ecosystem-based management implies a management scheme primarily designed to address overall ecosystem considerations”. Ecosystem-based management is seen as a governance instrument with an integrated approach that considers the structure and function of the entire ecosystem, including humans. The goal is to maintain healthy, resilient and productive ecosystems that can provide goods and services to the society (Braat and de Groot 2012). Often these MSP-supporting tools are innovative and seek to resolve the underlying user conflicts through better management of ocean areas and spaces by using, for instance, predictive models and trade-off analysis (Pınarbaşi et al. 2017)
Even though the ecosystem-based management has received considerable attention in recent years, there are still few demonstrations of its practical implementation (Murawski 2007; Curtin and Prelletzo 2010; Österblom et al. 2010; Tallis et al. 2010). One obstacle is that most marine monitoring and assessment programmes are sectoral, making it difficult to coordinate monitoring data and knowledge across sector programmes at the operational level (Grip and Olovsson 1990; Ottersen et al 2011)

### Ecological concept and principles

Ecological concepts, for example, levels of biological organization, native species, ecological resilience and disturbances are general understandings about how ecosystems are functioning and possible ways for managing them (Vold and Buffett 2008).

Ecological concepts, such as diversity of native species, habitat diversity and heterogeneity, key species and connectivity, are tied to basic assumptions or principles about ecosystems and how they function (Vold and Buffett 2008; Foley et al. 2010; Long et al. 2015). These principles have recently been raised as a base for and to guide an ecosystem-based MSP and decision-making process that integrates ecological, socio-economic and institutional perspectives, for example, in the conservation of biodiversity.

Ecological concepts and principles are used in physical planning, for example, to argue for conservation of biodiversity and ecosystems in connection with exploitation of marine resources.

When applied in concert with social, economic and governance principles, these ecological concepts and principles can support a more balanced management of activities in the oceans and seas (Fig. 1). This to maintain or restore healthy ecosystems, allow delivery of marine ecosystem services, and ensure sustainable economic and socio-cultural benefits. In that respect, natural and social sciences need to be better coordinated to improve compliance with the increasing needs (Foley et al. 2010; Gall and Rodwell 2016). The instrument for making this coordination possible is an overall ecosystem-based MSP process (Foley et al. 2010).

### Marine information and data management

Ecological and environmental sciences, including conservation biology, can provide the planner with necessary knowledge on environmental conditions, as a basis for sustainable use and management of natural resources and ecosystem services (Naturvårdsverket 2012). In that context, it is increasingly necessary to link knowledge from the physical and biological realms to social and economic drivers and their consequences to provide planners and decision-makers (politicians) with proper background information for solving problems (Mooney et al. 2013).

**Fig. 2:**
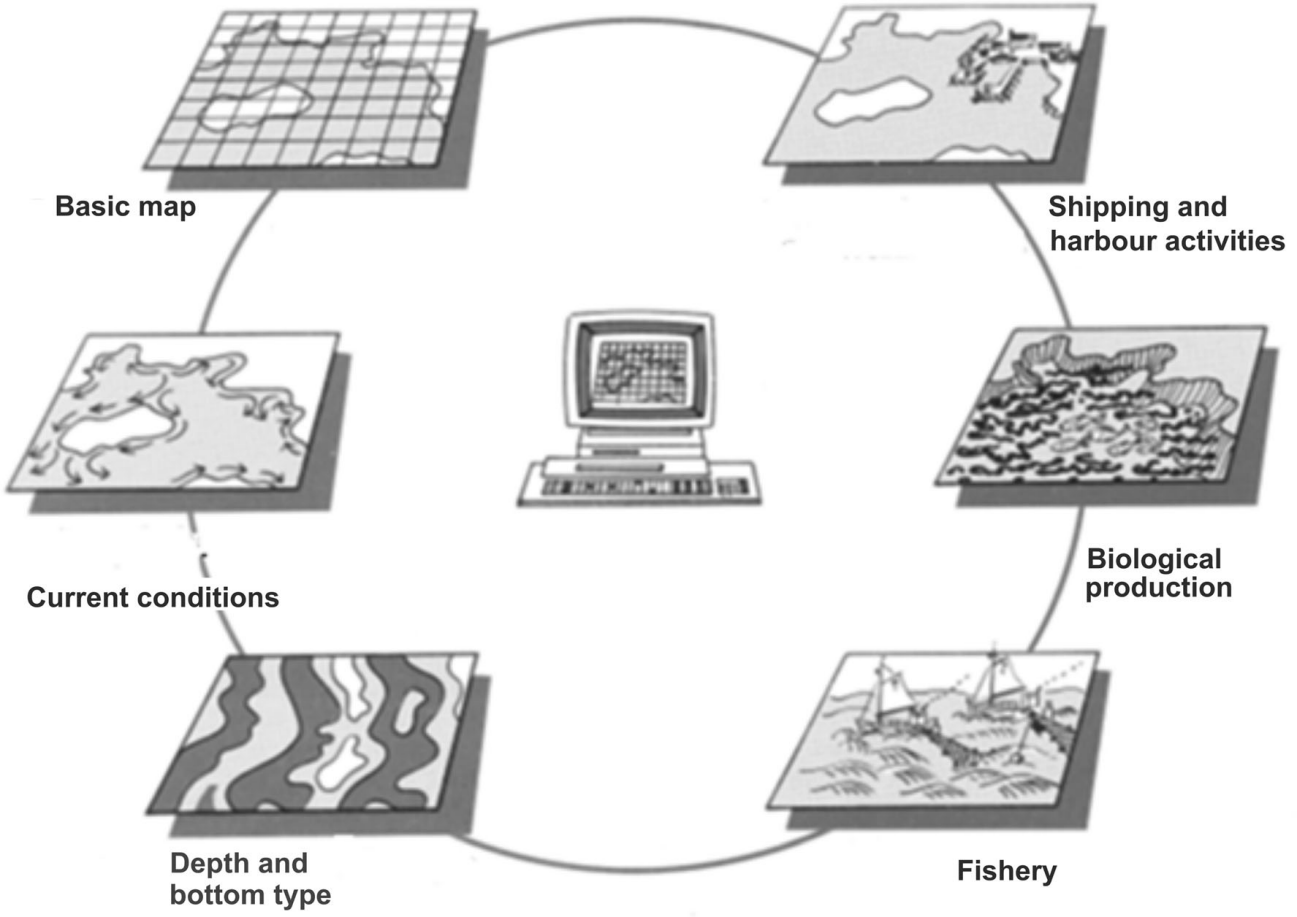
Marine information database for physical planning and natural resource management. *Source* Malmöhus County Administrative Board, Sweden (Linfo-kust 1990)

This means access to current and reliable information about important natural structures, and thus the conditions for the economic, ecological and socio-cultural values involved in the planning process (Fig. 2). Usually, the result leads to the formulation of different types of plans providing an overview and prioritization between different user interests (Ehler 2014; Santos et al. 2019).

As pressures on coastal and marine waters and their resources increase, it becomes more and more important to have access to adequate information about the qualities and values of these resources and ecosystem services, as well as about the different claims being made on them and the legislations involved. Data and knowledge regarding ecological, social and economic conditions need to be collected as part of a continuous planning process involving all relevant public authorities at local, regional, national and international levels (DSH 1989; Smythe 2017; Belgrano 2018).

What qualifies as a web-based decision support tool can range from simple records of data to complex systems for storing and processing data. In addition to central marine authorities, regional authorities and municipalities, there are several other bodies, which work with web-based marine data, e.g. at universities and colleges, governmental and non-governmental organizations (Merrifield et al. 2013).

Generally, such databases are constructed for a specific purpose only, within an authority or research institution. Opportunities for integration and interaction with other databases are rarely considered. This leads to difficulties in rationally gathering marine-oriented information for coastal and marine spatial planning. However, web-based marine data should be an open-source spatial tool that integrates and illuminates the human dimensions of marine science and policy (Merrifield et al. 2013).

## Implications of different pressures

Oceans and Seas are under increasing pressure from the effects of a steadily increasing human population, and increased use of resources. In addition, political systems and inadequate management schemes, in many cases, make it more difficult to respond to this increased pressure. Sources of environmental and social stress will likely increase in the future, not least related to the increasingly clear effects of climate change. The existing institutions and structures responsible for the coordination of the national marine environmental policies are in many cases too fragmented, and deal with the problems as sectoral issues (Grip 2017). There is an inability to manage present problems in a coherent and coordinated manner, and current development strategies will likely increase the vulnerability of the marine ecosystems and societies to these problems (Van Lavieren et al 2011; Dayton 2020).

The awareness of the importance of the oceans and seas challenges the traditional sector division and geographical limits in marine policy and raises the need for better coordinated and coherent marine policies.

In marine governance and management today, there is a move towards an explicit consideration of the effects of all marine sectors on the marine environment and its ecosystems. This comes from an increasing awareness of the cumulative effects of all human activities on the ecosystems. It has become obvious that there is a need to take a more holistic, multi-sectoral and coordinated, if possible integrated, approach to the management of marine usage and protecting the sustainability of marine ecosystems (O’Boyle and Jamieson 2006; Tallis et al. 2010; Ottersen et al. 2011).

Borja et al. (2016) stresses that human activities, both established and new, increasingly affect the provision of marine ecosystem services that provide societal and economic benefits. This influence has resulted in a need to incorporate and consider ecosystem functions and services along with the effects of all relevant human activities in the MSP process, while acknowledging that environmental assessments always have some uncertainty (Hammar 2014). Also, cross-boundary effects related to international agreements and implications at the political and institutional levels need to be considered.

Marine environmental governance and management is a national responsibility but will only be effective if nations have proper governance and management structures (e.g. institutions and legislation) in place, and collectively act responsibly through global and regional marine conventions and agreements (Hassler et al. 2018). The instrument for needed interaction and overall sectoral coordination between different socio-economic activities, environmental concerns, relevant stakeholders, concerned agencies and regulations is a participatory and overall ecosystem-based planning process in support of decision-making in marine management (Crowder and Norse 2008; Douvere 2008; Foley et al. 2010; Guerry et al. 2012; Carneiro 2013).

As mentioned, there are a number of ecological concepts and principles used in ecosystem-based planning and management and there is a tendency to focus on the environmental part of the planning process. However, the overall MSP process is a broader applicable tool for coordinating different marine interests and balancing the use, protection and conservation of marine areas and space with its resources, biodiversity and ecosystem services. In this regard, the MSP process would benefit from the participation of professional planners together with sectoral experts.

The basic aim of a multi-sectoral and ecosystem-based MSP process is to provide the decision-maker with a plan containing reliable information and proposals with guidelines for how the concerned interests should be distributed and managed. In that respect and in line with Faludi (1973), “it is the planner that is drawing up a plan or alternative plans on possible ways forward. However, the final decision is taken by the decision-maker, not the planner”.

This MSP process, which today is promoted globally, must not be bound to a specific sector. Therefore, it should be made clear which national body has the overall mandate to make the final decision. Otherwise, sector-bound planning and decision-making will continue to create management problems. Also, once the overall planning and decision-making procedure is over, it is the sectoral authorities and laws that forms the basis for the implementation and control according to the agreed plan.

Finally, an efficient integrative/coordinating MSP process needs to be anchored in a proper governance system, traditions and local knowledge, and have a long-term management perspective. It should be based on natural and socio-economic analyses of different users, include monitoring and evaluation aspects, promote cross-border cooperation and involve stakeholders and the general public in the process (Ehler 2017).

## Conclusions

The national administrative and legal system related to MSP varies between countries. However, the major characteristics of an overall ecosystem-based MSP process in support of coastal and marine decision-making can be summarized as follows:

- It is a multi-sectoral and overall planning process, concerned with managing the use and protection of coastal/marine resources and ecosystem services;
- It is a planning process in accordance with an MSP law, relevant sectoral laws and international conventions and agreements;
- It is a planning process responsible for granting of permits and other controls exercised by central and/or local government authorities;
- It is a planning process in which central and sometimes regional and local government bodies have been given a wide range of responsibilities for the implementation and control of decisions made by governmental agencies or the Government;
- It involves a continuous dialogue between different authorities at central, regional and local level in response to the requirements of different interests related to the use and protection of coastal and marine areas and space;
- It involves the coordination of different sectoral interests and their respective jurisdictions;
- It involves the participation of stakeholders, including non-governmental organizations’, in the planning process;
- It leads to an overall plan, with maps and regulations with guidelines, to ensure that coastal and marine resources are to be used in a manner commensurate with their carrying capacity. Also, taking into account the specific location of each resource and the requirements of the public;
- It includes the development of scientific knowledge about the ecosystem effects of activities related to the use of coastal and marine resources and ecosystem services, about the qualities and values of these resources and services, as well as the different claims on them;
- It aims for a consensus between coordinated interests (bodies). If consensus cannot be reached, the government, or an agency mandated by it, is responsible for the coordination of competing activities. This mandate also involves the responsibility for the final decision on how the water area and space should be used; and finally
- Once the decision is taken, the sectoral bodies are responsible for its implementation, enforcement and control, in accordance with the decided plan.

## Abbreviations

EU: European Union
IOC: International Oceanographic Commission
MARPOL: International Convention for the Prevention of Pollution from Ships
MSP: Marine Spatial Planning
UN: United Nations
UNCLOS: United Nations Convention on Law of the Sea
UNEP: United Nations Environment Programme
UNESCO: United Nations Educational, Scientific and Cultural Organization

## Footnotes

1 Maritime refer to industries/livelihoods related to the human use of ocean and sea resources, involving shipping, fishery, defense, energy, tourism, design, construction work, etc. (EU 2011).

2 A change may be under way. In December 2017, the General Assembly decided to convene an Intergovernmental Conference on an international legally binding instrument under the United Nations Convention on the Law of the Sea. The conference deals with the conservation and sustainable use of marine biological diversity of areas beyond national jurisdiction (General Assembly Resolution 72/249). The conference will meet in four sessions. The first session was held in September 2018, the second and third sessions were convened in 2019, while the fourth session initially planned for spring 2020 has been postponed.

3 The primary function of the International Seabed Authority is to regulate exploration for, and exploitation of deep seabed minerals found in ‘the Area’ according to UNCLOS. The International Seabed Authority should abide by an MSP regulation for the High Seas.
